# Engagement, Acceptability, Usability, and Preliminary Efficacy of a Self-Monitoring Mobile Health Intervention to Reduce Sedentary Behavior in Belgian Older Adults: Mixed Methods Study

**DOI:** 10.2196/18653

**Published:** 2020-10-29

**Authors:** Sofie Compernolle, Greet Cardon, Hidde P van der Ploeg, Femke Van Nassau, Ilse De Bourdeaudhuij, Judith J Jelsma, Ruben Brondeel, Delfien Van Dyck

**Affiliations:** 1 Department of Movement and Sport Sciences Ghent University Ghent Belgium; 2 Amsterdam UMC Vrije Universiteit Amsterdam Amsterdam Netherlands; 3 Research Foundation Flanders Brussels Belgium

**Keywords:** mHealth, older adults, self-monitoring, perceptions, engagement, acceptability, mixed methods

## Abstract

**Background:**

Although healthy aging can be stimulated by the reduction of sedentary behavior, few interventions are available for older adults. Previous studies suggest that self-monitoring might be a promising behavior change technique to reduce older adults’ sedentary behavior. However, little is known about older adults’ experiences with a self-monitoring–based intervention aimed at the reduction of sedentary behavior.

**Objective:**

The aim of this study is to evaluate engagement, acceptability, usability, and preliminary efficacy of a self-monitoring–based mHealth intervention developed to reduce older adults’ sedentary behavior.

**Methods:**

A mixed methods study was performed among 28 community-dwelling older adults living in Flanders, Belgium. The 3-week intervention consisted of general sedentary behavior information as well as visual and tactile feedback on participants’ sedentary behavior. Semistructured interviews were conducted to explore engagement with, and acceptability and usability of, the intervention. Sitting time was measured using the thigh-worn activPAL (PAL Technologies) accelerometer before and after the intervention. System usage data of the app were recorded. Quantitative data were analyzed using descriptive statistics and paired-samples *t* tests; qualitative data were thematically analyzed and presented using pen profiles.

**Results:**

Participants mainly reported positive feelings regarding the intervention, referring to it as motivating, surprising, and interesting. They commonly reported that the intervention changed their thinking (ie, they became more aware of their sedentary behavior) but not their actual behavior. There were mixed opinions on the kind of feedback (ie, tactile vs visual) that they preferred. The intervention was considered easy to use, and the design was described as clear. Some problems were noticed regarding attaching and wearing the self-monitoring device. System usage data showed that the median frequency of consulting the app widely differed among participants, ranging from 0 to 20 times a day. No significant reductions were found in objectively measured sitting time.

**Conclusions:**

Although the intervention was well perceived by the majority of older adults, no reductions in sitting time were found. Possible explanations for the lack of reductions might be the short intervention duration or the fact that only bringing the habitual sedentary behavior into conscious awareness might not be sufficient to achieve behavior change.

**Trial Registration:**

ClinicalTrials.gov NCT04003324; https://tinyurl.com/y2p4g8hx

## Introduction

The aging population continues to expand rapidly. Estimates indicate that the global number of adults over the age of 65 years will nearly double from a current population of about 800 million to approximately 1.5 billion in 2050 [1]. This unprecedented population boom poses a major public health challenge. Aging will present an economic burden on society because of increased needs resulting from age-related decline of physical, mental, and cognitive health [2]. To maintain the quality of life of older adults while living independently, healthy aging has become a main priority in the field of public health.

Up until now, the majority of efforts to facilitate healthy aging have been focused on increasing moderate- to vigorous-intensity physical activity [3] but have neglected sedentary behavior. However, both physical inactivity and high levels of sedentary time have been shown to be significantly related to detrimental health effects, like an increased risk for all-cause mortality, noncommunicable diseases [4], and geriatric syndromes, such as physical and cognitive impairments [5,6]. Research has indicated that an increase in moderate- to vigorous-intensity physical activity is often not sufficient to offset the negative health consequences of high levels of sedentary behavior [7]. Given the negative health consequences and the high prevalence of sedentary behavior in older adults (ie, 60 years and over) [8], creating interventions specifically focusing on the reduction of sedentary behavior is recommended to promote healthy aging.

Existing sedentary behavior interventions have mainly focused on social-cognitive models of behavioral change (eg, theory of planned behavior) [9,10]. However, most of these models are based on an expectancy-value framework in which behavior is determined by expected outcomes and the value that is placed on them [11]. As such, these models do not adequately capture processes underlying unintentional and habit-like behavior. Given that a large part of older adults’ sedentary behavior is habitual, specific strategies are needed to better control sedentary behavior. One might, for example, change the circumstances, so that habit cueing does not occur anymore [12], or alter external cues that lead to habit execution [13]. These strategies are rather manipulative and often impossible; therefore, they are not always ethical [14,15]. Another way to disrupt undesired habits is preferred, namely by bringing habitual behavior and its context into conscious awareness. This might be achieved by means of self-monitoring [16].

Self-monitoring, which is defined as keeping a record of a specified behavior as a method for changing behavior [16], has been identified as a promising behavior change technique to reduce sedentary behavior in adults [10,17]. A recent meta-analysis, in which interventions including self-monitoring were summarized that aimed to reduce sedentary behavior, showed a significant reduction in total sedentary time [17]. Specifically, an overall mean difference of 34.37 min/day (95% CI 14.48-54.25) was found for total sedentary time between intervention and control groups. It is important to note, however, that the majority of the included interventions targeted young and middle-aged adults. Only four studies targeted older adults with a mean age above 60 years. Of these four studies, only one used an electronic self-monitoring device to provide information on older adults’ sedentary behavior, namely the Fitbit One. As the Fitbit One is worn on the wrist, the validity of the sedentary behavior information can be questioned.

Given the limited quantity and quality of existing research on this topic, it remains unclear how older adults experience and use self-monitoring–based mobile health (mHealth) interventions specifically developed to reduce sedentary behavior. However, this information is essential to inform decisions on the development of future interventions. The conceptual model by Perski et al has indicated that user engagement (ie, the combination of subjective experiences characterized by attention, interest, and affect, and objectively measured intervention usage) is assumed to moderate the influence of the mHealth intervention on the mechanisms of action [18]. Next to user engagement, other aspects of acceptability (ie, how well older adults perceived the intervention and the extent to which the intervention met their needs), such as perceived relevance, satisfaction, and perceived usefulness, as well as usability (ie, the extent to which the intervention could be used by other older adults to reduce their sedentary behavior), also contribute to an individual’s motivation to continue using the app [19].

Therefore, the overall aim of this study is to gain insight into older adults’ experiences with, and the use of, a self-monitoring–based mHealth intervention specifically developed to reduce sedentary behavior. As both qualitative and quantitative data are essential to fully understand concepts such as user engagement, a mixed methods study is used. Moreover, preliminary efficacy of the intervention on older adults’ objectively assessed sedentary behavior is examined to get a first indication of the effect size.

## Methods

### Participants and Design

A convergence model with a triangulated mixed methods approach was conducted to gain in-depth comprehension in the user engagement, acceptability, and usability of an mHealth intervention aimed at the reduction of sedentary behavior. This methodology allowed us to compare, corroborate, or relate quantitative data (ie, system usage and activity monitor data) and qualitative data (ie, interview data). Quantitative and qualitative data were analyzed separately, followed by an integrated interpretation of the results. Participants in the mixed methods study were recruited in Flanders, Belgium, from February to May 2019 using convenience sampling. Recruitment continued until data were saturated (ie, until no new themes emerged in additional interviews). Firstly, an advertisement was distributed via Facebook, and secondly, the advertisement was electronically sent to older adults who were included in a previous study by our research group and who had expressed interest in future studies. To be eligible for this study, participants needed to (1) be at least 60 years old, (2) be Dutch speaking, (3) be able to walk 100 meters without severe difficulties, and (4) have a smartphone. The study was registered at ClinicalTrials.gov (Identifier: NCT04003324) and was approved by the Committee of Medical Ethics of the Ghent University Hospital (Belgian registration number: 2019/0398). All participants provided written informed consent.

### Procedure

**Figure 1:**
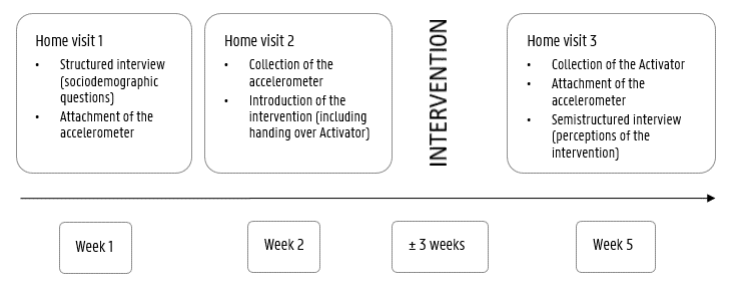
Study procedure.

The study procedure is explained in Figure 1. Concretely, older adults who agreed to participate were contacted by phone to make an appointment for a first home visit. During this home visit, they received an information letter explaining the purpose of the study and an informed consent form. After signing the informed consent form, baseline measures were collected. Specifically, a structured interview was conducted to assess participants’ sociodemographic characteristics. Moreover, an accelerometer—activPAL (PAL Technologies)—was attached to the participants’ thighs to objectively measure their sedentary behavior. Participants were instructed to wear the accelerometer for 1 week and to fill out the accompanying diary. After 1 week, a researcher visited the participants again at their homes to collect the accelerometers. After baseline measurements, the self-monitoring–based mHealth intervention was introduced to the participants (see Self-Monitoring mHealth Intervention section). At the end of the intervention (ie, after 3 weeks), participants were asked to complete a semistructured interview that included questions on user engagement with the intervention and perceptions regarding usability and acceptability. At the end of this last home visit, participants were instructed to wear the accelerometer for another week (ie, postmeasurements). Participants were given a prestamped envelope and were asked to send the accelerometer back by postal mail.

### Self-Monitoring mHealth Intervention

**Figure 2:**
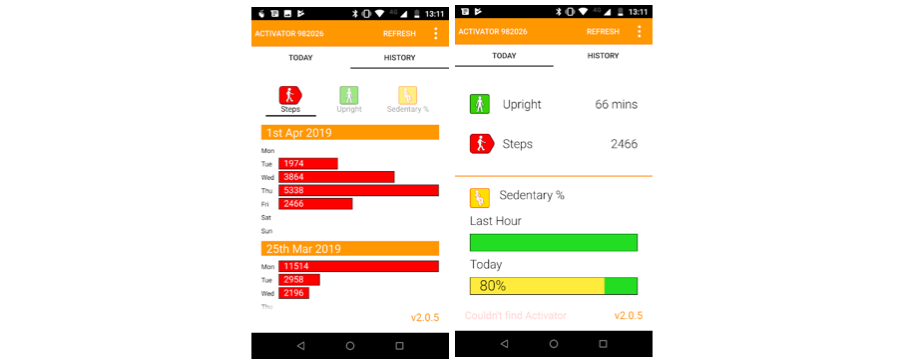
Screenshots of visual feedback provided by the Activator.

The intervention consisted of general sedentary behavior information as well as visual and tactile feedback on participants’ sedentary behavior. General sedentary behavior information was provided to participants by means of a 10-minute presentation. The presentation was given by an expert in the field during the second home visit. Visual and tactile feedback were provided using a novel self-monitoring device—the Activator (PAL Technologies). The Activator has recently been validated by Gill et al [20]. The Activator is worn on the front of the thigh, either in a pants pocket or attached with an elastic band to clothing covering the upper thigh (eg, trousers, jeans, shorts, leggings, tights, or dresses), and provides visual and tactile feedback [21]. Visual feedback is presented through a smartphone app via Bluetooth connection. Both real-time feedback and a 7-day historical overview are presented based on participants’ sedentary time, upright time, and number of steps (see Figure 2). Visual feedback is constantly available and can be viewed whenever and as often as participants want. Tactile feedback is provided by means of a strong, but comfortable, vibration of the Activator device itself each time a participant is sitting for 30 uninterrupted minutes. If a participant remains sedentary, the vibration is repeated after another 30 minutes. Participants were able to turn the vibration function on and off.

### Measurements

#### Structured Interview

Participants’ sociodemographic characteristics were collected by a trained researcher during the first home visit. Sociodemographic characteristics included age, gender, family situation (ie, being single or a widow or widower, having a partner but living independently, living with a partner, or being married), number of children, number of grandchildren, residential area (ie, countryside, village, city suburb, or city), educational level (ie, no education, primary education, vocational secondary education, technical secondary education, general secondary education, college, or university), and employment status (ie, employed or not employed).

#### Activity Monitor

Total sedentary time, sit-to-stand transitions, standing time, and number of steps were objectively estimated by means of the activPAL accelerometer. The accelerometer was attached on the midline of the right anterior thigh. Participants were instructed to wear the accelerometer for 7 consecutive days (24 h/day), both at baseline and at postmeasurement. The activPAL accelerometer summarizes data in 15-second intervals and has shown to be a valid and reliable measure for estimating the time spent sitting, standing, and stepping [19]. The activPAL data were downloaded using activPAL3 software, version 7.2.38, and were then processed using ProcessingPAL, version 1.1 (University of Leicester, UK). This software uses a validated algorithm to separate valid waking wear data from sleep and nonwear data. A day was considered invalid if there was limited postural variation (ie, ≥95% of wear time in one activity), a limited number of steps (<500 steps/day), or fewer than 10 hours of valid waking wear time [22,23]. Summary data from the algorithm were quality checked using heat maps against participants’ diaries, and corrections were made where needed [22,23]. Only participants with at least 5 days of valid activPAL data on both time points were included in the analyses [24].

#### Diary Log

Participants were asked to indicate sleep time (ie, time they went to bed and got up) and nonwearing time of the activPAL in a diary during the 7 days of baseline measurement and postmeasurement.

#### Semistructured Interview

Semistructured face-to-face interviews were conducted by trained researchers to explore (1) user engagement with the intervention and (2) the usability and acceptability of the intervention. User engagement was defined as the subjective experience of older adults with the intervention characterized by attention, interest, and affect. Acceptability was assessed by asking questions on how well the older adults perceived the intervention and by evaluating the extent to which the intervention met their needs. Usability included questions on the extent to which the intervention could be used by other older adults to reduce their sedentary behavior. The interview guide (see Multimedia Appendix 1) was developed by the first author (SC) based on an extensive literature search and on previous research by our research group examining user engagement, acceptability, and usability of eHealth and mHealth interventions [25]. Conceptual frameworks identified from the literature search, such as the conceptual framework of direct and indirect influences on engagement with digital behavior change interventions (DBCIs) by Perski et al [18] and the behavioral intervention technology (BIT) model of Mohr et al [26], guided the construction of the interview guide. The DBCI-related framework is an integrative conceptual framework involving potential direct and indirect influences on engagement and relationships between engagement and intervention effectiveness. The BIT model conceptually defines BITs, from the clinical aim to the technological delivery framework. 

After thorough discussion with the last author (DVD), the interview guide was revised and pilot-tested with two older adults. Based on the pilot test, some minor changes were made, such as paraphrasing and simplifying some vocabulary. By doing so, the clarity of the questions was verified and the duration of the interview was estimated. Interviews were audio recorded (mean duration 11.11 minutes, SD 5.94) and transcribed verbatim, producing a document of 122 pages in length, using Calibri font, size 11.

#### System Usage Data

System usage data of the Activator (ie, the app) were stored on the cloud server of PAL Technologies and used to objectively estimate user engagement; data included (1) the number of days the Activator was worn and (2) the number of times the app was accessed.

### Data Analysis

Descriptive statistics were used to describe the baseline characteristics of the participants and to assess the extent of usage (ie, engagement). Qualitative data were thematically analyzed, using the NVivo 12 software package (QSR International), using the six-phase approach by Braun and Clarke [27] to gain insight into participants’ subjective experiences with the intervention (ie, engagement) and acceptability and usability of the intervention. More specifically, two researchers outside the project team—Charlotte Meersseman and Siel Mechelinck—read and reread the transcripts multiple times to become familiar with the data (phase 1). They independently coded the data line by line and defined an initial coding scheme using an inductive approach (phase 2). The coding schemes were then discussed with the first author (SC). By doing so, the triangulation technique was applied and the trustworthiness and validity of the findings were promoted. Based on the coding schemes, themes were searched (phase 3), reviewed (phase 4), and defined (phase 5). Subsequently, pen profiles (ie, diagrams of composite key emergent themes, frequency data, and verbatim quotes) were constructed based on the defined themes and results were written up (phase 6). This increasingly utilized technique is considered appropriate for presenting qualitative outcome data in a clear and useful manner [28]. Paired-samples *t* tests were performed to determine preliminary efficacy of the intervention on older adults’ sedentary time. All quantitative analyses were conducted in SPSS Statistics for Windows, version 25.0 (IBM Corp).

## Results

### Participants

A total of 36 older adults expressed interest in participation. Out of these 36 participants, 2 (6%) of them could not be reached to make an appointment for the first home visit and 4 (11%) decided to withdraw from the study after receiving detailed study information. Reasons for withdrawal were health problems (2/36, 6%), lack of time (1/36, 3%), and death of a spouse (1/36, 3%). As such, 30 older adults completed the baseline measurements. Out of these 30 participants, 2 (7%) of them were excluded, as baseline data showed that they did not fulfill the inclusion criteria (ie, they were not able to walk 100 meters without severe difficulties). In addition, 2 (7%) participants dropped out during the intervention period due to health problems (1/30, 3%) and lack of motivation (1/30, 3%). Consequently, posttest data were collected from 26 out of 28 participants (93% retention).

Baseline characteristics of the participants are presented in Table 1. Just over half of the participants (15/28, 54%) were female, the average age was 65.0 years (SD 4.6), and the mean BMI was 25.4 kg/m^2^ (SD 3.9). The majority of the participants were highly educated and were married or lived with a partner.

**Table 1:** Baseline characteristics of the participants.

Characteristic		Values (N=28)
**Gender, n (%)**		
	Men	13 (46)
	Women	15 (54)
**Age in years**		
	All adults, mean (SD), range	64.3 (3.8), 60-76
	Young older adults (<65 years), n (%)	15 (54)
	Older adults (≥65 years), n (%)	13 (46)
**Educational level, n (%)**		
	No education or primary education	1 (4)
	Secondary education	11 (39)
	College or university	16 (57)
**Family situation, n (%)**		
	No partner (ie, single or widowed)	5 (18)
	Have a partner but living separately	1 (4)
	Married or living with a partner	22 (79)
**BMI**		
	Mean (SD)	25.4 (3.9)
	Healthy weight, n (%)	16 (57)
	Overweight, n (%)	6 (21)
	Obese, n (%)	5 (18)

### User Engagement

**Figure 3:**
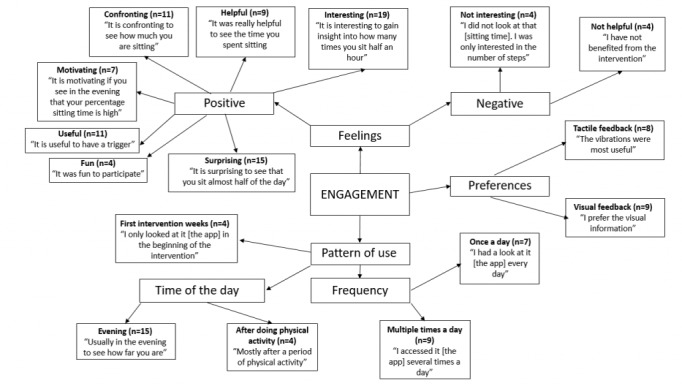
Pen profile of engagement with the intervention.

Qualitative data on user engagement were thematically analyzed and are presented in Figure 3. The main themes that emerged were positive and negative feelings about the intervention, preferences for the kind of feedback, and the pattern of use.

**Figure 4:**
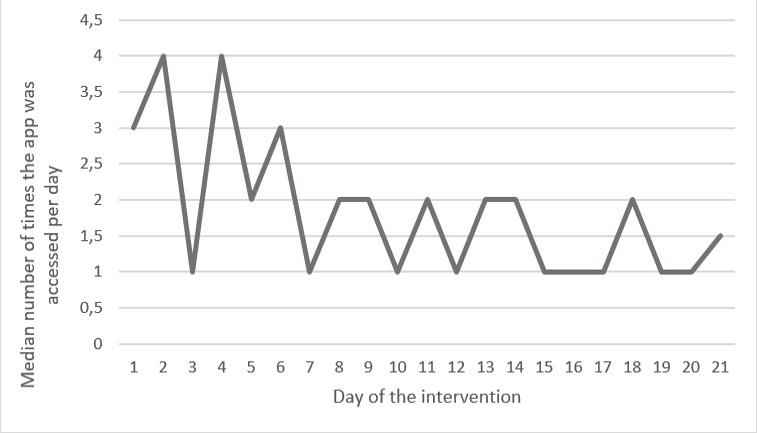
Evolution of consulting visual feedback.

The participants mainly reported positive feelings, such as being motivated, surprised, and interested. Only a minority (3/28, 11%) indicated that they thought the intervention was not interesting and not helpful. There were mixed opinions on the preferred kind of feedback (ie, tactile vs visual). Some thought the vibrations were more useful, whereas others favored the visual information on the app. System usage data showed that the median number of days the self-monitoring device was worn by the participants was 20 out of 21 days (range 15-21). Half of the participants (16/28, 57%) reported that they accessed the app on a daily basis. This finding was confirmed by system usage data (see Multimedia Appendix 2), showing that 8 out of 28 participants (29%) consulted the app every day, while 5 participants (18%) consulted the app at least 80% of the days. Some participants reported that they consulted the visual feedback multiple times a day. Accordingly, system usage data showed that the median frequency of consulting the app ranged from 0 to 20 times a day (see Multimedia Appendix 2). Especially in the evening, and after doing physical activities, participants reported that they viewed the visual feedback. They indicated that the main reasons to access the app were out of curiosity, to go through their day, and to see the impact of certain physical activities. Participants also emphasized that they consulted the app more frequently in the beginning of the intervention period, compared to the end of the intervention period. This finding was also in line with the system usage data, which show that the median frequency of consulting the app ranged from 3 or 4 times a day in the beginning of the intervention to 1 or 2 times a day at the end of the intervention (see Figure 4).

### Acceptability and Usability

**Figure 5:**
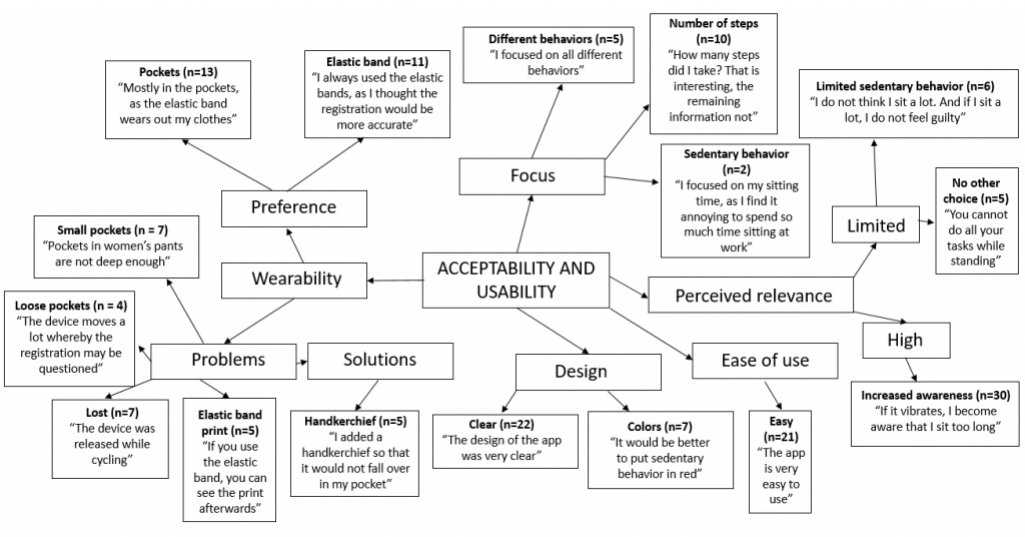
Pen profile on acceptability and usability of the intervention.

Results of the thematic analysis on acceptability and usability of the intervention are presented in Figure 5. The main themes that were identified were the design and the ease of use, wearing preferences, problems and solutions, the focus, and the perceived relevance. The intervention was considered easy to use, and most participants described the design as clear. The only remark on the design were the colors of the behaviors. Participants frequently cited that it would be more logical if sedentary behavior (ie, the behavior that should be limited) were displayed in red and the number of steps (ie, the behavior that should increase) in green. Participants expressed mixed preferences regarding the way to wear the device. Some participants (11/28, 39%) preferred to use the elastic band, whereas others (13/28, 46%) preferred to wear it in their pockets. Frequent problems that older adults, especially women, experienced included small or loose pockets, loss of the device, and the imprint of the elastic band on their clothes after wearing it. Out of the 28 older adults, 5 (18%) indicated that they used a handkerchief to ensure that the device was fixed in their pocket and could not flip over. Despite the fact that the aim of the intervention was to reduce sedentary behavior, only 2 participants (7%) mainly focused on the sedentary behavior information. Although the majority of older adults rated the intervention as highly relevant, some older adults were not convinced about the relevance. The most important reasons for limited perceived relevance were (1) the fact that they do not spend a lot of time sitting, or at least think they are not sitting a lot, and thus have no need to reduce their sedentary time and (2) the fact that they often see no other option but sitting to perform certain tasks.

### Preliminary Efficacy

Participants commonly reported that the intervention changed their thinking (ie, they became more *aware* of their sedentary behavior) but not their actual sedentary behavior. The latter result was supported by quantitative data derived from the activity monitor (see Table 2). Sitting and standing time were very similar at pre- and postmeasurements. There was a small improvement in steps of around 400 per day. This improvement was not significant, probably because of the small sample size.

**Table 2:** Preliminary efficacy of the intervention.

Variable	Values (N=26)		
	Baseline	Postintervention	*P* value
Total sedentary time (h/day), mean (SD)	8.7 (1.9)	8.8 (2.0)	.91
Sit-to-stand transitions (times per day), mean (SD)	50.2 (7.2)	50.4 (10.0)	.92
Standing time (h/day), mean (SD)	4.7 (1.3)	4.7 (1.3)	.76
Stepping time (h/day), mean (SD)	2.0 (0.8)	2.2 (0.8)	.58
Number of steps	4786	5193	.50

## Discussion

### Principal Findings

This study provides novel and in-depth insights into the potential of a self-monitoring–based mHealth intervention in older adults to reduce sedentary behavior. Overall, our results indicated that the intervention was generally well perceived by older adults, but preliminary analyses showed no reduction in sedentary time after the 3-week intervention period.

Previous research has shown that building sustained user engagement over time is challenging in mHealth interventions [29]. Low user engagement results in limited exposure to the intervention and, in turn, small or no intervention effects [30]. Therefore, gaining insight into the user engagement of mHealth interventions is crucial. Both objective usage data and subjective experiences showed that older adults were highly engaged with this study’s intervention. The participants generally expressed positive feelings, and the majority consulted the feedback frequently. They all agreed that the intervention made them more aware of their sedentary behavior, but the intervention did not result in a decrease in sedentary time.

The lack of a decrease in sedentary time is not entirely surprising given the following reasons. Firstly, the intervention period of 3 weeks was probably too short to actually change habitual behavior. Changing habits takes a long time [31] and, thus, it is likely that participants still need the cues to interrupt and/or reduce their sedentary behavior after the intervention has ended. Ending the cues might have resulted in relapse into their old and unhealthy habitual sedentary behavior [32]. Secondly, the intervention mainly targeted automatic processes underlying sedentary behavior by bringing the habitual behavior into conscious awareness. However, dual-process theories of motivation posit that both controlled and automatic processes regulate our sedentary behavior [33]. Thus, additional behavior change techniques (eg, goal setting, action planning, and coping planning) should be included in the intervention to affect the controlled processes and to actually achieve behavior change. Given that participants often mentioned that they saw no other options to reduce their sedentary behavior, it might be worth including concrete examples on how to reduce sedentary behavior. Thirdly, physical activity information (ie, number of steps) was also provided in the app, notwithstanding that the only aim of this intervention was to reduce sedentary behavior. The physical activity information could not be removed from the Activator app before the start of the study. Existing literature has indicated that participants of interventions targeting both sedentary behavior and physical activity simultaneously are more likely to focus on increasing physical activity due to (1) the clearer guidelines for physical activity (ie, 150 minutes of moderate- to vigorous-intensity physical activity a week) compared to sedentary behavior (ie, sit less), (2) the better-known negative health consequences of too little physical activity compared to too much sedentary behavior, and (3) the fact that physical inactivity is often still considered a synonym for sedentary behavior [34]. The latter was confirmed by the results of the semistructured interviews: participants often mentioned that they wanted to increase the number of steps in order to reduce their sedentary time. Objective physical activity data showed that the average daily number of steps increased by approximately 400, or 10%, over the 3-week intervention period. Although this increase was not significant, this indicates that Activator feedback is more likely to affect the number of steps than the sedentary time. This finding is in line with the results of previous Activator studies [21,35] and suggests that more efforts should be made to clarify the difference between sedentary behavior and physical inactivity and to emphasize the importance of standing and light-intensity physical activity.

Despite the fact that common aging-related barriers (eg, visual impairment, reduced working memory, limited motivation, and reduced mobility) can influence the use of mHealth in older adults [36], general perceptions on the acceptability and usability of this study’s intervention were positive. The app was easy to use and the design was clear. This is of great importance, as previous research has indicated that simplicity is one of the key principles for the design of mHealth interventions for older adults [37-39]. Although the Activator could be worn in different ways (ie, in the pants pockets or with an elastic band), the wearing of the device was often mentioned as challenging, especially among women. More research is therefore required to determine the ideal manner of attaching and wearing the Activator, especially when wearing pants without pockets or without deep pockets.

### Strengths and Limitations

Strengths of this study include the innovativeness of the research. To our knowledge, this is the first study examining older adults’ experiences with an electronic self-monitoring device specifically developed to reduce sedentary behavior. Moreover, by collecting both qualitative and quantitative data, a comprehensive view was obtained on self-monitoring–based mHealth interventions to reduce older adults’ sedentary behavior. An important limitation of this study was the sampling method. Participants were not randomly selected and, therefore, selection bias may have occurred. The majority of the participants were highly educated, whereby generalization of the results to lower-educated groups might be limited. Moreover, no control group was included, as the main aim was to gain in-depth knowledge on participants’ perceptions with the intervention. Although data saturation was achieved in the qualitative analysis, the small sample size was only sufficient to get a first indication on effect sizes and was not meant to provide sufficient statistical power for the quantitative analysis. Finally, the intervention lasted only 3 weeks and, thus, no conclusions can be drawn on the long-term adherence to the intervention. Based on these limitations, future studies should endeavor to recruit a larger, more generalizable sample and should use a randomized controlled trial design to draw firm conclusions on the effectiveness of a self-monitoring tool to reduce older adults’ sedentary behavior. Furthermore, we believe that adding behavior change techniques to the mHealth intervention, ones that can affect the controlled processes underlying sedentary behavior, and extending the intervention duration might be recommended in future studies.

### Conclusions

Results of this study suggest that the innovative self-monitoring–based mHealth intervention holds potential for the reduction of sedentary behavior in older adults. The intervention was considered interesting, helpful, and easy to use, and was able to increase awareness among older adults of their sedentary behavior. Despite the positive perceptions, no reductions in objective sedentary time were found in this study’s sample. Hence, the intervention was probably of insufficient intensity to reduce the sedentary behaviors of participants. In order to effectively achieve behavior change, a number of modifications to the intervention are suggested, such as the addition of behavior change techniques that target controlled processes underlying sedentary behavior.

## Appendix

Multimedia Appendix 1 Interview guide.

Multimedia Appendix 2 Frequency of app consultation per participant.

## Abbreviations

BIT: behavioral intervention technology
DBCI: digital behavior change intervention
mHealth: mobile health
