# Supplementary material for: Engagement, Acceptability, Usability, and Preliminary Efficacy of a Self-Monitoring Mobile Health Intervention to Reduce Sedentary Behavior in Belgian Older Adults: Mixed Methods Study
Source: JMIR Mhealth Uhealth. 2020 Oct 29;8(10):e18653. doi: 10.2196/18653 (PMC7661260; doi:10.2196/18653)
Supplement: Multimedia Appendix 1 [file mhealth_v8i10e18653_app1.docx]

Multimedia Appendix 1: Interview guide

Perceptions regarding self-monitoring sedentary behavior

- In general, how did you experience receiving personal sedentary behavior information?
- To which extent has receiving personal sedentary behavior helped you to reduce your sedentary behavior?
- To which extent has receiving personal sedentary behavior helped you to become more aware of your sedentary behavior?
- Which feedback (visual or tactile) did you prefer? Why?
- How often have you consulted the visual feedback?
- When have you consulted the visual feedback?
- What made you consult the visual feedback? What hindered you to consult the visual feedback?
- Which other strategies would helpful to reduce your sedentary behavior?

Perceptions regarding the acceptability and usability of the intervention

- How did you experience the app that provided sedentary behavior information?
- Was it easy to open the app? Which problems did you experience?
- What did you find about the visual feedback? Was it useful? If not, which information would be more useful? If yes, which information did you prefer?
- Do you feel the visual feedback was correct? If not, which activities were not correctly displayed?
- What did you think about the lay-out and the design of the app? What would you change?
- How did you perceive the technical assistance of the researchers?
- How did you perceive the self-monitoring tool?
- How did you experience attaching the Activator? Have you experiences problems? If yes, which problems? Any suggestions to solve these problems?
- How did you experience wearing the Activator? Have you experience inconveniences? How did you mostly wear the Activator? Why?
- Any additional comments?
