# Supplementary material for: Engagement, Acceptability, Usability, and Preliminary Efficacy of a Self-Monitoring Mobile Health Intervention to Reduce Sedentary Behavior in Belgian Older Adults: Mixed Methods Study
Source: JMIR Mhealth Uhealth. 2020 Oct 29;8(10):e18653. doi: 10.2196/18653 (PMC7661260; doi:10.2196/18653)
Supplement: Multimedia Appendix 2 [file mhealth_v8i10e18653_app2.docx]

Multimedia Appendix 2: Frequency of consulting the app per participant
